# Analysis of medical services provided to patients with peripheral facial palsy in Korea: a descriptive, cross-sectional study of the health insurance review and assessment service national patient sample database

**DOI:** 10.1186/s12913-021-07078-9

**Published:** 2021-10-29

**Authors:** Doori Kim, Boyoung Jung, Myoung-Ui Cho, Seong-Bae Song, Seol Hee Chung, Tae-Yong Park, In-Hyuk Ha

**Affiliations:** 1 Jaseng Spine and Joint Research Institute, Jaseng Medical Foundation, 3F 538 Gangnam-daero, Gangnam-gu, Seoul, 06110 South Korea; 2 Department of Health Administration, Hanyang Women’s University, 200, Salgoji-gil, Seongdong-gu, Seoul, 04763 South Korea; 3 Bucheon Jaseng Hospital of Korean Medicine, 191 Buil-ro, Wonmi-gu, Bucheon, 14598 Republic of Korea; 4 Department of Research, Health Insurance Review & Assessment Service, 60 Hyeoksin-ro (Bangok-dong), Wonju-si, Gangwon-do 26465 South Korea; 5 Institute for integrative medicine, Catholic Kwandong University International St. Mary’s Hospital, Incheon, South Korea

**Keywords:** Bell’s palsy, Facial palsy, Healthcare services, Korean traditional medicine, Korean health insurance review and assessment national patient sample

## Abstract

**Background:**

This cross-sectional, retrospective, observational study analyzed the demographics of patients with peripheral facial palsy in South Korea and their use of healthcare services.

**Methods:**

The 2016 Korean Health Insurance Review and Assessment National Patient Sample dataset was used; a total of 4790 patients, diagnosed with facial palsy, who had used healthcare services at least once between January 2016 and December 2016 were included, and data on the use of medical services, hospitalizations, sociodemographic factors, treatments, and medications were analyzed.

**Results:**

Overall, 326 patients per 100,000 individuals used healthcare services at least once because of peripheral facial palsy in 2016, with higher numbers for women and those aged 50–59 years. The percentage of patients who used Korean traditional medicine (KM), Western medicine (WM), and both KM and WM was 54.4, 23.3, and 22.3%, respectively. Users of both WM and KM had higher per capita medical costs, more visits, and longer treatment durations. Physiotherapy was the most frequent WM treatment (44.4%), and “examinations” was the costliest (24.7%) category. “Procedures” was both the most frequent and costliest KM category (99.9 and 57.3%, respectively). “Continuous intravenous injections” (8.6%) and “superficial heat therapy” (8.3%) were the most frequent WM treatments, while acupuncture accounted for 98% of all KM treatments.

**Conclusions:**

This study analyzed the demographic characteristics and medical service use of patients with peripheral facial palsy in detail. These results can be used as basic information to improve clinical and policy strategies for the management and treatment of peripheral facial palsy.

**Supplementary Information:**

The online version contains supplementary material available at 10.1186/s12913-021-07078-9.

## Background

The seventh cranial nerve, also known as the facial nerve, is important because it allows people to convey facial expressions by enabling the voluntarily contraction of facial muscles. It also controls taste sensations in the anterior two-thirds of the tongue [1]. Idiopathic facial palsy, or Bell’s palsy, is a type of facial palsy that results in dysfunction of the facial nerve on the affected side. The most common type of the disease is acute mononeuropathy of an unknown cause, characterized by rapid partial or complete unilateral facial palsy [2, 3].

Approximately 71% of patients with peripheral facial palsy recover with no sequelae; however, the remaining 29% experience various sequelae, of which 4% are serious [4]. Although peripheral facial palsy is not a life-threatening disease, it leads to psychological distress and a reduced quality of life [5–15] because the face is, from a psychological perspective, the most important human body part [16].

However, despite these difficulties faced by patients with peripheral facial palsy, studies on the specific medical services used are scarce. Furthermore, although it has been reported that the annual incidence rate of facial palsy ranges from 11.5 to 40.2 per 100,000 individuals [17], the data on the demographic characteristics remain unclear [4, 18, 19]. Despite the clinical guidelines regarding the treatment of facial palsy being relatively well established, there is insufficient information about the use of specific medical services and the current state of treatments other than those involving standard Western medicine (WM). Indeed, many patients with peripheral facial palsy in Southeast Asia use Korean traditional medicine (KM) or Chinese medicine.

In particular, South Korea has a dichotomized healthcare system that covers WM and KM [20, 21], and facial palsy is one of the diseases frequently treated by using a combination of these approaches, ranking sixth on the outpatient and tenth on the inpatient ranking list for combined Western and Korean medical treatment [22, 23]. Analyzing the current state of treatments for facial palsy in Korea is thus highly informative because it enables an examination of multiple aspects of the current treatment patterns.

Therefore, in light of the lack of detailed information about peripheral facial palsy, especially in the context of the dichotomized healthcare system in South Korea, this study aimed to use the Health Insurance Review and Assessment National Patient Sample (HIRA-NPS), a large-scale administrative dataset that represents the general South Korean population, to investigate the current state of patients with facial palsy, with a special focus on comparing the use of WM and KM.

## Methods

This was a cross-sectional, retrospective, observational study that analyzed the 2016 HIRA-NPS data. The study was approved by the Institutional Review Board of Jaseng Hospital of Korean Medicine in Seoul, South Korea (JASENG 2018–09-006). It was not possible to obtain patient consent because encrypted and published data were used in this study.

South Korea has a national health insurance system that insures approximately 98% of the population. It covers the service costs of both WM and KM and can account for 30–‍50% of total personal medical costs. Claims are created when healthcare providers file for the reimbursement of provided healthcare services, and about 46 million claims cases are filed every year. Claims data contain useful information on patient diagnosis, received care, surgery, drugs, and healthcare providers, although access to researchers used to be limited because of the vast volume of data. Consequently, the HIRA established the “Patient Sample” dataset to increase access to claims data. This dataset, created through a random stratified sampling of HIRA claims data according to sex and age [24], is representative of the South Korean population; it is updated annually, and the number of samples approximates 1.4 million, which is about 3% of the entire population [25].

Previous studies defined peripheral facial palsy solely on the basis of the 10th revision of the International Statistical Classification of Disease and Related Health Problems code G51.0 (Bell’s palsy) or by including the codes G51.1 (geniculate ganglionitis), G51.2 (Melkersson’s syndrome) [26], G51.4 (facial myokymia), G51.8 (other disorders or facial syndromes), and G51.9 (unspecified facial nerve disorder)—all of these definitions have been validated [27, 28]. In this study, facial palsy was defined based on codes G51.x, although patients who were diagnosed using two relatively less common codes, G51.1 and G51.2 (*N* = 17 and *N* = 1, respectively), were excluded. Furthermore, since two consulting neurologists suggested that G51.4 is generally not assigned to facial palsy, patients diagnosed with G51.4 were also excluded.

Accordingly, patients who used health services at least once in 2016 and were listed under code G51.0, G51.8, or G51.9 were included in the analysis (Table S1). The weighting values were the same (33.33) and thus were not taken into consideration.

The general use of medical services, prevalence of facial palsy per 100,000 individuals, and demographic characteristics were analyzed according to the use of outpatient/inpatient services and the type of medical service received (KM/WM/both). Patients who received inpatient care at least once were counted as inpatients, and those who received outpatient care at least once were counted as outpatients, thus permitting duplicate counting of patients. Patients who only used KM for facial palsy treatment were defined as KM users, and those who used only WM for its treatment were defined as WM users. Those who used both KM and WM were defined as users of both.

We analyzed demographic characteristics according to sex and age, which was divided into decades: < 20 years, 20–29 years, 30–39 years, 40–49 years, 50–59 years, 60–69 years, and ≥ 70 years.

We also analyzed the patients’ visits to various healthcare facilities, which were classified according to the common medical institutions in South Korea, including general, primary care, and tertiary hospitals, and KM clinics and hospitals. The frequency of use of the different medical specialties was also assessed; less frequently used specialties were classified as “others.” Duplicate counting of the same patient was permitted when the patient visited multiple types of healthcare facilities and specialty departments.

The medical-cost-per-service category was derived from the claims data and the corresponding mean medical cost per service and mean medical cost per bill were separately computed for KM and WM claims. Medical costs were defined as the total medical care expenses, including copayments and insurance coverage, and were classified according to the categories provided by the Ministry of Health and Welfare. This classification defines 10 categories for WM (consultations, hospitalizations, medication, injections, anesthesia, physiotherapy, psychotherapy, procedures/surgery, examinations, and radiographic evaluations/interventions) and five categories for KM (consultations, hospitalizations, medication, procedures, and examinations) [29]. The costs for psychotherapy were excluded as they rarely occurred. To assess the number of claims that were filed per category, the number of claims were counted, not the number of patients. All claims and all relevant medical costs were included in the total number of claims medical costs.

We also analyzed data on received care, which included injections, procedures, physical therapy, and medication—Table S2 shows the specific codes that were used. WM and KM were analyzed separately; WM was classified into injections; nerve blocks; and physical therapy, which was further classified into heat, electrical, rehabilitation, and other forms of therapy, whereas KM was classified into acupuncture, electric acupuncture stimulation, moxibustion, cupping, meridian warming therapy, and examinations. Treatment data were obtained based on the corresponding codes, as opposed to limiting one patient to one claims bill, thus permitting duplicate counting of patients regarding the type of care and procedures received.

The drug prescription status was analyzed for both inpatients and outpatients. The analysis was based on the frequency at which medications were prescribed, excluding digestants. The Anatomical Therapeutic Chemical Classification System, a drug classification system developed by the World Health Organization Collaborating Centre for Drug Statistics Methodology in 1976 [30, 31], involves five levels, each corresponding to major anatomical groups, major therapeutic groups, therapeutic/pharmacological subgroups, chemical/therapeutic/pharmacological subgroups, or chemical substances. In this study, drugs were described as chemical substances, the fifth level of this system. Only three drugs (mecobalamin, sodium hyaluronate, and loxoprofen sodium), which lack the fifth-level code, were listed using their generic names. Each drug code was counted, allowing duplicate counting for the same patient when one patient was prescribed multiple drugs.

The general medical service use and expenses, according to users of KM, WM, or both, and outpatient/inpatient status are presented as means and standard deviations (SDs). The prevalence of facial palsy, patient demographic characteristics, number of bills, and medical costs per category are presented as frequencies and percentages. Other medical service-related details, including data on the healthcare facility visited, medical specialty, WM treatment details (injection treatments, nerve blocks, physical therapy), KM treatment details (acupuncture, electric acupuncture stimulation, moxibustion, cupping, etc.), and prescribed medicine are presented as frequencies and percentages according to the inpatient/outpatient status and users of KM, WM, or both. All data processing and analyses were conducted using the SAS package (version 9.4; SAS Institute Inc., Cary, NC, USA).

## Results

### General use of medical services among patients with facial palsy

Table 1 shows the use of general medical services by patients with facial palsy in 2016. A total of 4790 patients accessed medical services at least once for facial palsy. The medical expense per capita was $395 (SD 933), the average number of days of treatment was 15.8 (SD 28.3), and the average number of visits was 14.2 (SD 25.0). Regarding the type of medical service, 2608 (54.4%) patients only used KM, 1114 (23.3%) only used WM, and 1068 (22.3%) used both KM and WM. The medical expense per capita was $961 (SD 985) for users of both types of medical care, which was higher than that for users of KM or WM alone. Furthermore, the number of days of treatment and visits were higher in users of both types of medical care. The number of users of outpatient and inpatient services were 4690 and 519, respectively, showing that only 10.8% of the entire pool of patients received inpatient care (Table 2). The medical expenses per capita, average number of visits, and average number of days of treatment were higher among patients receiving both KM and WM for both the outpatient and inpatient service user groups.

**Table 1:** General medical service use and expenses for patients with facial palsy

	Number of patients (%)	Total expense^**a**^	Expense per patient^**a**^		Number of visits^**b**^		Days of treatment^c^	
			Mean	SD	Mean	SD	Mean	SD
**Total**								
Total	4790 (100)	1,894,107	395	933	14.2	25.0	15.8	28.3
KM user	2608 (54.4)	517,616	198	357	10.4	18.9	10.8	19.4
WM users	1114 (23.3)	349,960	314	1453	5.3	20.1	7.8	29.5
Users of both	1068 (22.3)	1,026,531	961	985	32.8	32.5	36.3	34.9
**Outpatients**^d^								
Total	4690 (100)	1,271,710	271	445	13.2	22.6	14.2	25.2
KM users	2631 (56.1)	911,425	346	344	10.6	19.0	11.0	19.5
WM users	1057 (22.5)	120,011	114	168	3.6	8.1	5.3	21.6
Users of both	1002 (21.4)	635,995	635	638	30.1	31.1	31.9	32.7
**Inpatients**^**e**^								
Total	519 (100)	622,397	1199	2141	12.1	28.2	17.5	31.3
KM users	107 (20.6)	98,024	916	674	12.5	10.2	12.6	10.3
WM users	324 (62.4)	370,645	1144	2595	9.6	33.8	16.6	37.7
Users of both	88 (17.0)	153,728	1747	1153	21.0	15.8	26.6	17.6

*KM* Korean traditional medicine, *WM* Western medicine, *SD* standard deviation

^a^Expense of items determined to be eligible for reimbursement by the HIRA (Health Insurance Review and Assessment Service) out of the total treatment amount was indicated in the submitted insurance claim statement. This was converted to U.S. Dollars on October 12, 2018 (USD 1.00 = South Korean Won 1130)

^b^Number of visits: Days of visits to actual medical institutions such as hospitals

^c^Days of treatment: Number of treatment days, including drug prescription days

^d^Outpatients: Patients with at least one outpatient visit

^e^Inpatients: Patients who used inpatient services at least once

**Table 2:** Prevalence of facial palsy and patient demographic features according to Korean traditional medicine services used

	Prevalence^a^	Total					
		KM users		WM users		Users of both	
		N	%	N	%	N	%
**Total**	326	2608		1114		1068	
**Sex**							
Male	262	848	32.5	484	43.5	545	51.0
Female	388	1760	67.5	630	56.6	523	49.0
**Age**							
< 20	44	40	1.5	48	4.3	44	4.1
20–29	128	88	3.4	78	7.0	71	6.7
30–39	220	196	7.5	139	12.5	135	12.6
40–49	304	359	13.8	185	16.6	197	18.5
50–59	501	704	27.0	239	21.5	255	23.9
60–69	687	600	23.0	229	20.6	206	19.3
≥ 70	729	621	23.8	196	17.6	160	15.0

*KM* Korean traditional medicine, *WM* Western medicine

^a^Prevalence: Prevalence per 100,000 individuals. Number of samples in the 2016 National Patient Sample is 1,468,033. Prevalence per 100,000 individuals was calculated as (Total number)*100,000/1,468,033

Moreover, the use of various medical facilities and specialties among patients was also analyzed (Table S4). Most patients visited a Korean medical clinic (*N* = 3390, 70.8%). Regarding the medical specialty, most outpatients frequented internal Korean medicine departments (*N* = 2199, 46.9%), while most inpatients visited neurology departments (*N* = 135, 26.0%).

### Distribution of medical expenses by category among patients with facial palsy

Table 3 shows the distribution of medical expenses by category among patients with facial palsy. Regarding WM bills, 61,417 (90.2%) were for outpatient visits, followed by 4696 and 2434 bills listing physiotherapy (44.4%) and examinations (23.0%), respectively. Medical costs per category were the highest for examinations, accounting for 24.7% of all WM expenses, followed by those for hospitalizations (17.7%) and consultations (16.0%). Regarding KM bills, most were for procedures and outpatient visits (*N* = 51,934, 99.9% and *N* = 51,869, 99.8%, respectively)—medical costs were the highest for procedures (57.3%). Unlike for patients using WM, the medical costs related to examinations and hospitalizations for patients using KM were insignificant.

**Table 3:** Number of bills and medical costs per category

	Total					WM^**a**^					KM^**b**^				
	Case		Cost			Case		Cost			Case		Cost		
	N^c^	%^e^	Total^d^	%	Per case^d^	N^c^	%	Total^d^	%	Per case^d^	N^c^	%	Total^d^	%	Per case^d^
**Total**	62,586		1,894,107		30.3	10,588		740,607		69.9	51,998		1,177,452		22.6
**Outpatient visits (consultations)**	61,417	98.1	491,841	25.6	8.0	9548	90.2	119,028	16.1	12.5	51,869	99.8	372,813	31.7	7.2
**Hospitalizations**	550	0.9	247,027	12.9	449.1	360	3.4	130,765	17.7	363.2	190	0.4	116,263	9.9	611.9
**Medication**	7863	12.6	34,348	1.8	4.4	1054	10.0	22,232	3.0	21.1	6809	13.1	12,116	1.0	1.8
**Injections**	1495	2.4	27,906	1.5	18.7	1495	14.1	27,906	3.8	18.7	–	–	–	–	–
**Anesthesia**	433	0.7	13,636	0.7	31.5	433	4.1	13,636	1.8	31.5	–	–	–	–	–
**Physiotherapy**	4696	7.5	88,787	4.6	18.9	4696	44.4	88,787	12.0	18.9	–	–	–	–	–
**Procedure/surgery**	52,092	83.2	691,548	36.1	13.3	158	1.5	16,397	2.2	103.8	51,934	99.9	675,150	57.3	13.0
**Examinations**	2708	4.3	184,190	9.6	68.0	2434	23.0	183,081	24.7	75.2	274	0.5	1109	0.1	4.0
**Radiographic Evaluations/interventions**	624	1.0	11,422	0.6	18.3	624	5.9	11,422	1.5	18.3	–	–	–	–	–

^a^WM: Number of bills and medical costs per category for Western care

^b^KM: Number of bills and medical costs per category for Korean traditional medical care

^c^N: Total/Western/Korean medical bills

^d^The cost of items determined to be eligible for reimbursement by the HIRA (Health Insurance Review and Assessment Service) out of the total treatment amount was indicated in the submitted insurance claim statement. It was converted to U.S. Dollars on October 12, 2018 (USD 1.00 = South Korean Won 1130)

^e^%: N/total number of bills*100, percentage of bills containing the corresponding category

### Care for facial palsy

Data on WM treatment for facial palsy are presented according to inpatient/outpatient status and the use of either KM or WM (Table 4). Injection types, divided into continuous intravenous, subcutaneous or intramuscular, and intravenous side injections, were administered to 426 (8.6%), 285 (5.7%), and 151 (3.0%) patients, respectively. The percentage of injections was higher among inpatients than outpatients. More injection prescriptions were listed for users of both types of medical care than users of WM alone. Nerve blocks were classified into “blocks of peripheral branches of spinal nerves,” “sympathetic plexus or ganglion blocks,” “cranial nerve or peripheral branch blocks,” and “spinal nerve plexus, root or ganglion blocks,” and administered to 38 (0.8%), 36 (0.7%), 39 (0.8%), and 14 (0.3%) patients, respectively. The most frequent form of physical therapy was superficial heat therapy (*N* = 411, 8.3%), followed by electrical stimulation (*N* = 394, 7.9%) and massage therapy (*N* = 285, 5.7%). The trends in the frequency of use of physical therapies were similar between users of both types of medical care and users of WM alone, with superficial heat therapy, electrical stimulation therapy, and massage therapy being the most frequently used physical therapies overall. Users of both types of medical care generally received physical therapy more often than users of KM alone. Physical therapy rates were higher among inpatients than outpatients.

**Table 4:** Care for facial palsy—Western medicine treatment

	Total						Outpatients						Inpatients					
	Total		Users of both		WM users		Total		Users of both		WM users		Total		Users of both		WM users	
	N	%	N	%	N	%	N	%	N	%	N	%	N	%	N	%	N	%
	4970		1068		1114		4690		1002		1057		519		88		324	
**Injections**																		
Continuous intravenous injections	426	8.6	278	26.0	148	13.3	101	2.2	64	6.4	37	3.5	335	64.5	54	61.4	281	86.7
Subcutaneous or intramuscular injections	285	5.7	165	15.4	120	10.8	204	4.3	114	11.4	90	8.5	40	7.7	20	22.7	20	6.2
Intravenous side injections	151	3.0	97	9.1	54	4.8	33	0.7	22	2.2	11	1.0	121	23.3	18	20.5	103	31.8
**Nerve blocks**																		
Block of peripheral branch of spinal nerve-greater or lesser occipital nerve	38	0.8	13	1.2	25	2.2	36	0.8	12	1.2	24	2.3	2	0.4	0	0.0	2	0.6
Sympathetic plexus or ganglion block	36	0.7	23	2.2	13	1.2	35	0.7	22	2.2	13	1.2	3	0.6	0	0.0	3	0.9
Cranial nerve or its peripheral branch block-facial nerve	39	0.8	10	0.9	29	2.6	27	0.6	7	0.7	20	1.9	0	0.0	0	0.0	0	0.0
Spinal nerve plexus, root or ganglion block-superficial cervical plexus	14	0.3	1	0.1	13	1.2	14	0.3	1	0.1	13	1.2	0	0.0	0	0.0	0	0.0
**Physical therapy**																		
Superficial heat therapy	411	8.3	290	27.2	121	10.9	342	7.3	236	23.6	106	10.0	98	18.9	29	33.0	69	21.3
Deep heat therapy	188	3.8	128	12.0	60	5.4	147	3.1	100	10.0	47	4.4	47	9.1	15	17.0	32	9.9
Infrared ray irradiation	88	1.8	62	5.8	26	2.3	79	1.7	56	5.6	23	2.2	15	2.9	5	5.7	10	3.1
Electrical stimulation therapy	394	7.9	305	28.6	89	8.0	321	6.8	245	24.5	76	7.2	116	22.4	41	46.6	75	23.1
Transcutaneous electrical nerve stimulation	150	3.0	97	9.1	53	4.8	123	2.6	76	7.6	47	4.4	30	5.8	11	12.5	19	5.9
Interferential current therapy	65	1.3	40	3.7	25	2.2	42	0.9	25	2.5	17	1.6	24	4.6	13	14.8	11	3.4
Laser therapy	69	1.4	45	4.2	24	2.2	61	1.3	42	4.2	19	1.8	13	2.5	6	6.8	7	2.2
Massage therapy	285	5.7	216	20.2	69	6.2	240	5.1	179	17.9	61	5.8	73	14.1	23	26.1	50	15.4
Simple therapeutic exercise	80	1.6	61	5.7	19	1.7	66	1.4	49	4.9	17	1.6	17	3.3	5	5.7	12	3.7
Therapeutic exercise-complex	12	0.2	7	0.7	5	0.4	7	0.1	5	0.5	2	0.2	6	1.2	1	1.1	5	1.5
Myofascial trigger point injection therapy	10	0.2	4	0.4	6	0.5	8	0.2	4	0.4	4	0.4	2	0.4	0	0.0	2	0.6

*WM* Western medicine

Table 5 shows the details regarding KM care received by patients with facial palsy. Among 3676 patients who used KM (KM users alone and users of both types of medical care), 98.3% (3613) received acupuncture therapy. The prevalence was similar for outpatients and inpatients, with 98.3 and 99.5%, respectively, receiving acupuncture therapy. Regarding specific acupuncture therapies, both inpatients and outpatients most frequently underwent penetration needling. A total of 1506 (41.0%) patients received electric acupuncture stimulation, and 1359 (37.0%) received moxibustion therapy. A total of 1166 (31.7%) patients received dry cupping therapy, while a lower number (*N* = 1074, 29.2%) received wet cupping therapy. A total of 2398 (65.2%) patients received meridian warming therapy. Only a small percentage of patients underwent KM tests, with the percentage of patients undergoing the meridian function test, the most frequently used test, being 4.7% among outpatients and 9.23% among inpatients. Except for some specific acupuncture therapies, the percentage of patients undergoing KM therapies and tests was higher among inpatients than among outpatients.

**Table 5:** Care for facial palsy—Korean traditional medicine treatment

	Total		Outpatients^a^		Inpatients^**b**^	
	N	%	N	%	N	%
	3676		3633		195	
**Acupuncture therapy**						
**Basic acupuncture**	3613	98.3	3571	98.3	194	99.5
**Special acupuncture**						
Penetration needling	2676	72.8	2627	72.3	178	91.3
Intra-articular	849	23.1	844	23.2	7	3.6
Intervertebral	309	8.4	304	8.4	6	3.1
Orbital	273	7.4	257	7.1	29	14.9
Intra-abdominal	128	3.5	127	3.5	3	1.5
**Electric acupuncture stimulation**	1506	41.0	1477	40.7	123	63.1
**Moxibustion**	1359	37.0	1296	35.7	127	65.1
**Cupping**						
Wet cupping	1074	29.2	1033	28.4	77	39.5
Dry cupping	1166	31.7	1104	30.4	94	48.2
**Warming meridian**	2398	65.2	2373	65.3	145	74.4
**Tests**						
Meridian function test	188	5.1	172	4.7	18	9.2
Electro-pulse graph	41	1.1	31	0.9	11	5.6
Ryodoraku	18	0.5	16	0.4	3	1.5

^a^Outpatients: Patients with at least one outpatient visit

^b^Inpatients: Patients who used inpatient services at least once

### Drug prescriptions for facial palsy

Table 6 shows the most frequently prescribed drugs for patients with facial palsy. Adrenocortical hormones were both the first and second most prescribed drugs. The most frequently prescribed drug was prednisolone, which was prescribed to 16.0% (*N* = 750) of outpatients and 38.5% (*N* = 200) of inpatients. The second most frequently prescribed drug was methylprednisolone, prescribed to 3.8% (*N* = 176) of outpatients and 12.9% (*N* = 67) of inpatients. The third most frequently prescribed drug was the antiviral agent famciclovir, which was prescribed to 3.8% (*N* = 189) of all patients. Among the top 19 most frequently prescribed drugs, three were adrenocortical hormones and three were antiviral agents. Other frequently prescribed drugs included psychiatric (the psychotonics choline alfoscerate and acetylcarnitine, the sedatives diazepam and alprazolam, and the antidepressant amitriptyline) and analgesic drugs (paracetamol and loxoprofen sodium). The medication rate was higher for inpatients than for outpatients and higher for users of both types of medical care than for users of WM alone.

**Table 6:** Drug prescriptions for facial palsy

	Total						Outpatients^**a**^						Inpatients^**b**^					
	Total		Users of both		WM users		Total		Users of both		WM users		Total		Users of both		WM users	
	N	%	N	%	N	%	N	%	N	%	N	%	N	%	N	%	N	%
	4970		1068		1114		4690		1002		1057		519		88		324	
**Prednisolone**	852	17.1	607	56.8	245	22.0	750	16.0	529	52.8	221	20.9	200	38.5	34	38.6	166	51.2
**Methylprednisolone**	230	4.6	168	15.7	62	5.6	176	3.8	130	13.0	46	4.4	67	12.9	13	14.8	54	16.7
**Famciclovir**	189	3.8	145	13.6	44	3.9	150	3.2	119	11.9	31	2.9	47	9.1	12	13.6	35	10.8
**Mecobalamin**^c^	183	3.7	123	11.5	60	5.4	174	3.7	117	11.7	57	5.4	19	3.7	1	1.1	18	5.6
**Sodium hyaluronate**^**c**^	175	3.5	134	12.5	41	3.7	131	2.8	98	9.8	33	3.1	51	9.8	8	9.1	43	13.3
**Dexamethasone**	171	3.4	105	9.8	66	5.9	126	2.7	73	7.3	53	5.0	47	9.1	8	9.1	39	12.0
**Paracetamol**	131	2.6	85	8.0	46	4.1	89	1.9	54	5.4	35	3.3	49	9.4	11	12.5	38	11.7
**Valaciclovir**	116	2.3	81	7.6	35	3.1	93	2.0	67	6.7	26	2.5	31	6.0	4	4.5	27	8.3
**Aciclovir**	103	2.1	73	6.8	30	2.7	86	1.8	61	6.1	25	2.4	23	4.4	3	3.4	20	6.2
**Choline alfoscerate**	95	1.9	46	4.3	49	4.4	83	1.8	36	3.6	47	4.4	15	2.9	1	1.1	14	4.3
**Diazepam**	88	1.8	35	3.3	53	4.8	80	1.7	28	2.8	52	4.9	11	2.1	1	1.1	10	3.1
**Acetylsalicylic acid**	83	1.7	26	2.4	57	5.1	58	1.2	17	1.7	41	3.9	32	6.2	0	0.0	32	9.9
**Eperisone**	81	1.6	37	3.5	44	3.9	64	1.4	29	2.9	35	3.3	21	4.0	2	2.3	19	5.9
**Alprazolam**	76	1.5	35	3.3	41	3.7	61	1.3	23	2.3	38	3.6	16	3.1	3	3.4	13	4.0
**Loxoprofen sodium**^**c**^	73	1.5	36	3.4	37	3.3	69	1.5	34	3.4	35	3.3	4	0.8	1	1.1	3	0.9
***Ginkgo biloba***	71	1.4	33	3.1	38	3.4	62	1.3	25	2.5	37	3.5	9	1.7	2	2.3	7	2.2
**Acetylcarnitine**	59	1.2	31	2.9	28	2.5	45	1.0	25	2.5	20	1.9	19	3.7	3	3.4	16	4.9
**Amitriptyline**	49	1.0	25	2.3	24	2.2	37	0.8	18	1.8	19	1.8	14	2.7	1	1.1	13	4.0
**Clopidogrel**	44	0.9	12	1.1	32	2.9	32	0.7	5	0.5	27	2.6	18	3.5	0	0.0	18	5.6

*WM* Western medicine

^a^Outpatients: Patients with at least one outpatient visit

^b^Inpatients: Patients who used inpatient services at least once

^c^Anatomical Therapeutic Chemical fourth level medication

## Discussion

In this study, the prevalence of peripheral facial palsy in 2016 was 326 per 100,000 individuals, which was slightly higher than what has been reported in previous studies conducted at a national level (120–210 per 100,000 individuals) [5, 32]. This difference can be attributed to the fact that, whereas previous studies only included patients with peripheral facial palsy of House-Brackmann grade III or higher, this study included all patients who used healthcare services at least once due to peripheral facial palsy.

The prevalence of peripheral facial palsy was approximately 1.5 times higher in females (males: 262/100,000; females: 388/100,000). While this suggests that the prevalence of peripheral facial palsy, considering the definition used in our study, is generally higher among females, it may simply reflect the fact that females generally use healthcare services more frequently [33]. Since most previous studies do not report sex-specific differences in this regard [4, 17, 18], the latter explanation seems more convincing; nevertheless, further studies are needed to clarify this issue. In this study, the prevalence of peripheral facial palsy increased with age, which is in line with previous studies [5, 34] that also reported peripheral facial palsy not only being more common among older age groups [18, 19, 35], but also being associated with more sequelae because of lower complete recovery rates [4]. Furthermore, the number of patients who received KM treatments for peripheral facial palsy was three times higher than those who did not, and female and older patients, both inpatients and outpatients, used KM more often. This is in line with known preferences for KM in the general Korean population [36].

The average medical expenses and cost per bill were lower and the number of visits and days of treatment were higher for users of KM than for users of WM. This suggests that patients used KM more frequently and at a lower cost per treatment than WM. There are three possible explanations for this finding. First, users of KM may have been overtreated. Second, KM may have been ineffective, thereby lengthening the treatment duration. Finally, users of KM may have received appropriate and continuous care. Several studies have shown that KM is effective for peripheral facial palsy [37–47] and that several sessions are required to alleviate the symptoms: 90 sessions of acupuncture therapy showed 100% efficacy and a cure rate of 90% [48], and 20 sessions of acupuncture therapy improved serious sequelae in a patient with peripheral facial palsy 20 years after onset [49]. These findings support the third explanation—that patients have received appropriate and continuous care—however, there is a lack of high-quality randomized controlled trials on the effectiveness of KM and acupuncture for peripheral facial palsy, indicating the need for further studies [50, 51].

The most and the second most frequently prescribed drugs were glucocorticoids, their prescription rate being higher among users of both types of medical care than among users of WM alone. Glucocorticoids are the most frequently prescribed drug for patients with peripheral facial palsy [30] and, in general, their use is recommended for acute peripheral facial palsy treatment [52, 53]; however, oral steroid administration is associated with numerous side effects, including electrolyte disturbances, blood pressure elevation, hyperglycemia, pancreatitis, and other hematological, immunological, and neuropsychiatric problems [54, 55]. In this study, approximately 50% of the users of KM were not prescribed steroids for peripheral facial palsy. It is not clear whether this indicates that KM treatment for peripheral facial palsy may have a reduced steroid use, which highlights the need for further research. It was also found that psychotonics were used for peripheral facial palsy treatment (Table 6), suggesting the involvement of psychological factors [56].

This study had certain limitations. All the available medical care cost data for patients with peripheral facial palsy were not considered. In general, medical care costs are classified into direct, indirect, and intangible costs [57]; however, this study only analyzed a part of the direct costs, namely medical care costs directly resulting from healthcare services, thereby excluding non-medical costs such as those for transportation and caregivers. Furthermore, in South Korea, some procedures are not covered by national insurance; therefore, based solely on claims data, it cannot be assumed that all KM options for patients with peripheral facial palsy have been considered. Moreover, patients were identified according to the main diagnosis in their claim, which may undermine the accuracy of the data [58] because of different etiologies for peripheral facial palsy. In particular, chronic and acute peripheral facial palsy could not be distinguished. Although this issue was discussed at great length during the study design, we concluded that it was impossible to distinguish between chronic and acute peripheral facial palsy. However, the diagnostic codes were selected carefully through consultations between neurologists. Moreover, outcomes for patients with peripheral facial palsy could not be analyzed, and there was no comparison of the treatment effects between WM and KM.

It would be an interesting to compare the results of the present study with national representative data for patients with facial palsy from other countries. Further study is also necessary to analyze the demographic characteristics, treatment status, and treatment outcomes by distinguishing acute and chronic facial palsy.

Despite the limitations of the source data and the cross-sectional design, this study was the first to use health insurance claims data to investigate a sample of patients with peripheral facial palsy that most closely resembles the general population in South Korea. Furthermore, our focus remained on thoroughly investigating the treatment status of both known and unknown causes (Bell’s palsy) based on the purpose of the study. Considering the lack of studies comparing the KM and WM for patients with peripheral facial palsy, the present study is meaningful. It could provide objective information on the various treatments for peripheral facial palsy and medical costs. The results of the study would be meaningful for clinical specialists and policymakers.

## Conclusions

This study analyzed in detail the recent state and medical service use of patients with peripheral facial palsy in Korea. Specifically, it provided new information on the specific medical use and medical costs of patients with peripheral facial palsy based on a comparison between KM and WM treatments. In Korea, most peripheral facial palsy patients are treated with KM, and predominantly with acupuncture in KM; with regard to WM, prednisolone was used the most. The treatment method, period, and cost varied depending on the patients. These results can be used as a basis for improving clinical and policy strategies for the management and treatment of peripheral facial palsy.

## Supplementary Information

**Additional file 1: Table S1.** Patients who used healthcare services more than once according to the diagnostic codes for facial palsy.

**Additional file 2: Table S2.** Treatment codes and drug ingredient codes.

**Additional file 3: Table S3.** Prevalence of facial palsy and patient demographic features according to Korean traditional medicine services used.

**Additional file 4: Table S4.** Use of various healthcare facilities and medical specialties among patients with facial palsy.

## Data Availability

The datasets generated during and/or analyzed in the current study are available in the HIRA-NPS repository. The study utilized HIRA data, which are third-party data and thus not owned by the authors. The HIRA data are available upon direct request, via email or fax, and submission of the request form and declaration of data use, which are downloadable from the HIRA website [http://opendata.hira.or.kr/op/opc/selectPatDataAplInfoView.do], and upon payment of a data request fee (300,000 KRW per dataset).

## Abbreviations

HIRA-NPS: Health Insurance Review and Assessment National Patient Sample
KM: Korean traditional medicine
WM: Western medicine
