# Supplementary material for: Analysis of medical services provided to patients with peripheral facial palsy in Korea: a descriptive, cross-sectional study of the health insurance review and assessment service national patient sample database
Source: BMC Health Serv Res. 2021 Oct 29;21:1178. doi: 10.1186/s12913-021-07078-9 (PMC8555159; doi:10.1186/s12913-021-07078-9)
Supplement: Supplementary file 1 — Additional file 1: Table S1. Patients who used healthcare services more than once according to the diagnostic codes for facial palsy. [file 12913_2021_7078_MOESM1_ESM.docx]

Table S1. Patients who used healthcare services more than once according to the diagnostic codes for facial palsy

| **ICD** | **Name** | **N** | **%** |
| --- | --- | --- | --- |
| **G51.0** | Bell's palsy | 2,845 | 51.81 |
| **G51.1** | Geniculate ganglionitis | 17 | 0.31 |
| **G51.2** | Melkersson's syndrome | 1 | 0.02 |
| **G51.4** | Facial myokymia | 734 | 13.37 |
| **G51.8** | Other disorders or facial syndromes | 835 | 15.21 |
| **G51.9** | Disorder of facial nerve, unspecified | 1,811 | 32.98 |

ICD, International Classification of Disease.
