# Supplementary material for: Analysis of medical services provided to patients with peripheral facial palsy in Korea: a descriptive, cross-sectional study of the health insurance review and assessment service national patient sample database
Source: BMC Health Serv Res. 2021 Oct 29;21:1178. doi: 10.1186/s12913-021-07078-9 (PMC8555159; doi:10.1186/s12913-021-07078-9)
Supplement: Supplementary file 2 — Additional file 2: Table S2. Treatment codes and drug ingredient codes. [file 12913_2021_7078_MOESM2_ESM.docx]

Table S2. Treatment codes and drug ingredient codes.

| **Classification** | **EDI Code** | **Procedure/chemical ingredient** |
| --- | --- | --- |
| **Western medical care** | |  |
|  | KK051, KK052, K053 | Continuous intravenous injection |
|  | KK010 | Subcutaneous or intramuscular injection |
|  | KK054 | Intravenous side injection |
|  | la241 | Block of peripheral branch of spinal nerve-greater or lesser occipital nerve |
|  | la261 | Sympathetic plexus or ganglion block-simple |
|  | la232, la341 | Cranial nerve or its peripheral branch block-facial nerve |
|  | la251 | Spinal nerve plexus, root or ganglion block-superficial cervical plexus |
|  | mm010, mm015 | Superficial heat therapy |
|  | mm020, mm 015 | Deep heat therapy |
|  | mm300 | Infrared ray irradiation |
|  | mm060 | Electrical stimulation therapy |
|  | mm070 | Transcutaneous electrical nerve stimulation |
|  | mm080 | Interferential current therapy |
|  | mm085 | Laser therapy |
|  | mm090 | Massage therapy |
|  | mm101 | Simple therapeutic exercise |
|  | mm102 | Therapeutic exercise-complex |
|  | mm131 | Myofascial trigger point injection therapy |
| **Korean medical care** | |  |
|  | km4001 | Basic acupuncture |
|  | km4008 | Special acupuncture_Penetration needling |
|  | km4006 | Special acupuncture_Intraarticular |
|  | km4007 | Special acupuncture_Intervertebral |
|  | km4003 | Special acupuncture_Orbital |
|  | km4005 | Special acupuncture_Intraabdominal |
|  | km4009 | Electric acupuncture stimulation |
|  | km4030 | Moxibustion |
|  | km4031 | Wet cupping |
|  | km4032 | Dry cupping |
|  | km4070 | Warming the meridian therapy |
|  | km2003 | Meridian function test |
|  | km2002 | Electro pulse test |
|  | km2001 | Ryodoraku |
| **Drug ingredient codes** | |  |
|  | P217001AT | Prednisolone |
|  | P193302AT | Methylprednisolone |
|  | P157201AT | Famciclovir |
|  | P188602AT | Mecobalamin |
|  | P530020CO | Sodium hyaluronate |
|  | P142232BI | Dexamethasone |
|  | P101430AT | Paracetamol |
|  | P246701AT | Valaciclovir |
|  | P102904AT | Aciclovir |
|  | P138101AC | Choline alfoscerate |
|  | P142902AT | Diazepam |
|  | P111001AT | Acetylsalicylic acid |
|  | P152301AT | Eperisone |
|  | P105502AT | Alprazolam |
|  | P186101AT | Loxoprofen sodium |
|  | P165305AT | Ginkgo biloba |
|  | P101601AT | Acetylcarnitine |
|  | P107501AT | Amitriptyline |
|  | P136901AT | Clopidogrel |

EDI, electronic data interchange.
