# Supplementary material for: Analysis of medical services provided to patients with peripheral facial palsy in Korea: a descriptive, cross-sectional study of the health insurance review and assessment service national patient sample database
Source: BMC Health Serv Res. 2021 Oct 29;21:1178. doi: 10.1186/s12913-021-07078-9 (PMC8555159; doi:10.1186/s12913-021-07078-9)
Supplement: Supplementary file 3 — Additional file 3: Table S3. Prevalence of facial palsy and patient demographic features according to Korean traditional medicine services used. [file 12913_2021_7078_MOESM3_ESM.docx]

|  |  | **Outpatients**^*^ | | | | | | | **Inpatients^†^** | | | | | | | | |
| --- | --- | --- | --- | --- | --- | --- | --- | --- | --- | --- | --- | --- | --- | --- | --- | --- | --- |
|  |  | KM users | | WM users | | Users of both | | | KM users | | | WM users | | | Users of both | | |
|  | Prevalence^‡^ | N | % | N | % | N | % | N | | % | N | | % | N | | % |  |
| **Total** | 326 | 2,631 | | 1,057 | | 1,002 | | | 107 | | | 324 | | | 88 | | |
| **Sex** |  |  |  |  |  |  |  |  | |  |  | |  |  | |  |  |
| Male | 262 | 860 | 32.7 | 457 | 43.2 | 516 | 51.5 | 49 | | 45.8 | 161 | | 49.7 | 41 | | 46.6 |  |
| Female | 388 | 1771 | 67.3 | 600 | 56.8 | 486 | 48.5 | 58 | | 54.2 | 163 | | 50.3 | 47 | | 53.4 |  |
| **Age** |  |  |  |  |  |  |  |  | |  |  | |  |  | |  |  |
| <20 | 44 | 41 | 1.6 | 49 | 4.6 | 42 | 4.2 | 2 | | 1.9 | 14 | | 4.3 | 3 | | 3.4 |  |
| 20–29 | 128 | 91 | 3.5 | 76 | 7.2 | 67 | 6.7 | 4 | | 3.7 | 14 | | 4.3 | 5 | | 5.7 |  |
| 30–39 | 220 | 200 | 7.6 | 134 | 12.7 | 131 | 13.1 | 9 | | 8.4 | 39 | | 12.0 | 11 | | 12.5 |  |
| 40–49 | 304 | 361 | 13.7 | 174 | 16.5 | 190 | 19.0 | 14 | | 13.1 | 58 | | 17.9 | 10 | | 11.4 |  |
| 50–59 | 501 | 714 | 27.1 | 227 | 21.5 | 234 | 23.4 | 25 | | 23.4 | 77 | | 23.8 | 25 | | 28.4 |  |
| 60–69 | 687 | 604 | 23.0 | 217 | 20.5 | 193 | 19.3 | 24 | | 22.4 | 65 | | 20.1 | 21 | | 23.9 |  |
| ≥70 | 729 | 620 | 23.6 | 180 | 17.0 | 145 | 14.5 | 29 | | 27.1 | 57 | | 17.6 | 13 | | 14.8 |  |

Table S3. Prevalence of facial palsy and patient demographic features according to Korean traditional medicine services used

^*^Outpatients: Patients with at least one outpatient visit
**^†^**Inpatients: Patients who used inpatient services at least once
^‡^Prevalence: Prevalence per 100,000 individuals. Number of samples in the 2016 National Patient Sample is 1,468,033. Prevalence per 100,000 individuals was calculated as (Total number)*100,000/1,468,033.
KM, Korean traditional medicine; WM, Western medicine.
