# Supplementary material for: Analysis of medical services provided to patients with peripheral facial palsy in Korea: a descriptive, cross-sectional study of the health insurance review and assessment service national patient sample database
Source: BMC Health Serv Res. 2021 Oct 29;21:1178. doi: 10.1186/s12913-021-07078-9 (PMC8555159; doi:10.1186/s12913-021-07078-9)
Supplement: Supplementary file 4 — Additional file 4: Table S4. Use of various healthcare facilities and medical specialties among patients with facial palsy. [file 12913_2021_7078_MOESM4_ESM.docx]

Table S4. Use of various healthcare facilities and medical specialties among patients with facial palsy.

|  |  | **Total** | | **Outpatients^*^** | | **Inpatients^†^** | |
| --- | --- | --- | --- | --- | --- | --- | --- |
|  |  | N | % | N | % | N | % |
| **Medical institution type** | |  |  |  |  |  |  |
|  | Tertiary hospital | 479 | 10.0 | 447 | 9.5 | 110 | 21.2 |
|  | General hospital | 798 | 16.7 | 723 | 15.4 | 209 | 40.3 |
|  | Hospital | 290 | 6.1 | 257 | 5.5 | 54 | 10.4 |
|  | Primary care clinic | 948 | 19.8 | 943 | 20.1 | 8 | 1.5 |
|  | Long-term hospital | 52 | 1.1 | 24 | 0.5 | 30 | 5.8 |
|  | Korean medical clinic | 3,390 | 70.8 | 3,390 | 72.3 | 4 | 0.8 |
|  | Korean medical hospital | 467 | 9.7 | 422 | 9.0 | 152 | 29.3 |
| **Medical specialty** | |  |  |  |  |  |  |
|  | Neurology | 1,283 | 26.8 | 1,236 | 26.4 | 135 | 26.0 |
|  | Neurosurgery | 356 | 7.4 | 331 | 7.1 | 49 | 9.4 |
|  | Otorhinolaryngology | 250 | 5.2 | 235 | 5.0 | 61 | 11.8 |
|  | Rehabilitative medicine | 204 | 4.3 | 188 | 4.0 | 20 | 3.9 |
|  | Internal medicine | 187 | 3.9 | 173 | 3.7 | 19 | 3.7 |
|  | Internal Korean medicine | 2,232 | 46.6 | 2,199 | 46.9 | 60 | 11.6 |
|  | Acupuncture and moxibustion | 1,754 | 36.6 | 1,737 | 37.0 | 98 | 18.9 |
|  | Others | 699 | 14.6 | 574 | 12.2 | 192 | 37.0 |

^*^Outpatients: Patients with at least one outpatient visit
^†^Inpatients: Patients who used inpatient services at least once
